# Plant Water Use Efficiency over Geological Time – Evolution of Leaf Stomata Configurations Affecting Plant Gas Exchange

**DOI:** 10.1371/journal.pone.0067757

**Published:** 2013-07-02

**Authors:** Shmuel Assouline, Dani Or

**Affiliations:** 1 Department of Environmental Physics and Irrigation, Institute of Soil, Water and Environmental Sciences, A.R.O.-Volcani Center, Bet Dagan, Israel; 2 Department of Environmental Sciences (D-UWIS), Institute of Terrestrial Ecosystems (ITES), Soil and Terrestrial Environmental Physics (STEP), Swiss Federal Institute of Technology (ETH), Zurich, Switzerland; Estacion Experimental de Zonas Áridas (CSIC), Spain

## Abstract

Plant gas exchange is a key process shaping global hydrological and carbon cycles and is often characterized by plant water use efficiency (WUE - the ratio of CO_2_ gain to water vapor loss). Plant fossil record suggests that plant adaptation to changing atmospheric CO_2_ involved correlated evolution of stomata density (*d*) and size (*s*), and related maximal aperture, *a_max_*. We interpreted the fossil record of *s* and *d* correlated evolution during the Phanerozoic to quantify impacts on gas conductance affecting plant transpiration, *E*, and CO_2_ uptake, *A,* independently, and consequently, on plant WUE. A shift in stomata configuration from large *s-*low *d* to small *s-*high *d* in response to decreasing atmospheric CO_2_ resulted in large changes in plant gas exchange characteristics. The relationships between gas conductance, *g_ws_*, *A* and *E* and maximal relative transpiring leaf area, (*a_max_*⋅*d*), exhibited hysteretic-like behavior. The new WUE trend derived from independent estimates of *A* and *E* differs from established WUE-CO_2_ trends for atmospheric CO_2_ concentrations exceeding 1,200 ppm. In contrast with a nearly-linear decrease in WUE with decreasing CO_2_ obtained by standard methods, the newly estimated WUE trend exhibits remarkably stable values for an extended geologic period during which atmospheric CO_2_ dropped from 3,500 to 1,200 ppm. Pending additional tests, the findings may affect projected impacts of increased atmospheric CO_2_ on components of the global hydrological cycle.

## Introduction

Aspects of plant gas exchange operating at the stomatal scale may exert significant influences on global hydrological and carbon cycles with about 40% of terrestrial precipitation evaporating back to the atmosphere through plant leaves [1]–[6]. The extent of plant influence on hydrological and carbon cycles is directly linked with plant water use efficiency (WUE) defined as the ratio of CO_2_ gain by assimilation, *A* [mol L^−2^ T^−1^], to water vapor loss by transpiration, *E* [mol L^−2^ T^−1^]:
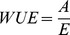
(1)


Stomata and impervious leaf cuticle have originated some 400 Myr ago as crucial adaptations enabling plant proliferation in water-limited terrestrial habitats [5], [7]–[9]. Plant CO_2_ uptake for photosynthesis and concurrent water loss by transpiration are dynamically regulated by stomata perforating leaf surfaces. The intertwined control of CO_2_ uptake and water loss obscures the primary driving process, prompting a century-long debate concerning the primary regulating factor [10], [11].

A widely-used approach for the evaluation of plant WUE in Eq. (1) is based on (i) estimating maximal water vapor diffusive leaf conductance, *g_w_* [mol m^−2^ s^−1^], considering diffusion through an ensemble of non-interacting individual pores [12]–[16]; and (ii) invoking similarity between water vapor conductance, *g_w_*, and CO_2_ conductance, *g_c_* [1], [12], [14], [15], [17]–[19], resulting in a convenient and widely used relation for gas conductance:
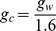
(2)


The factor 1.6 is the ratio of binary diffusivities of CO_2_ and water vapor in air. The gas fluxes based on Fickian diffusion are expressed as [11], [20]:
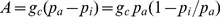
(3)

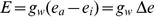
(4)with *p_i_* and *p_a_* are the leaf interior and atmospheric partial pressures of CO_2_; and similarly *e_i_* and *e_a_* are the leaf interior and atmospheric water vapor pressures, with Δ*e* = (*e_a_*–*e_i_*) often expressed in units of [mol gas mol^−1^ air]. For low respiration rates relative to *A*, Eqs. 3 and 4 are similar to the model of Von Caemmerer and Farquhar [21]. According to Eqs. 1–2–3–4, plant WUE is reduced to the simple expression [20], [22], [23]:
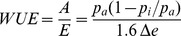
(5)where a constant 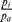 value is generally applied [20]. Tanner and Sinclair [24] reviewed water use in crop production and found a relatively constant value of 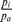 = 0.7 for C3 species. For relatively constant values of Δe and 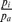, the resulting plant WUE based on Eq. 5 is proportional to atmospheric CO2 partial pressure, *p_a_*.

Noting that information concerning plant stomatal configuration cancels out in Eq. 5, we show in the following that considering the two key diffusion processes separately while accounting for the effects of specific stomatal configurations yields a significantly different relationship between plant WUE and CO_2_ that deviates from the simple linear expression in Eq. 5.

During the 400 million years (Myr) of plant evolution (the Phanerozoic eon), atmospheric CO_2_ concentrations (*CO_2_)* decreased by one order of magnitude from 4,000 ppm to present day levels [25]-[28]. The plant fossil record for the same period revealed that plant gas exchange adaptation involved significant variations in stomatal density, *d* [mm^−2^], and size (stomatal area), *s* [µm^2^] [14], [15], [29], [30]. Stomatal size, *s*, estimated by multiplying guard cell length by the width of guard cell pair [14], [15], is used to estimate stomatal maximal aperture, *a_max_*, approximated as 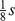 [14], [31]. The correlated evolution of *s* and *d* is in line with theoretical and experimental evidence demonstrating that stomata *a_max_* and *d* jointly determine gas diffusive resistance, a nonlinear function of these two variables [32]–[42].

We hypothesize that improved description of diffusive resistances by consideration of specific stomatal configurations deduced from plant fossil record could provide new insights on the independent responses of *A* and *E* to variations in atmospheric CO_2_ during the Phanerozoic. The primary objectives of this study were to: (i) analyze the impact of independent estimates of *A* and *E* on historical trends in plant WUE relative to the coupled estimates based on Eq. 5; and (ii) infer the role of stomata configuration evolution on plant gas exchange and WUE estimates during the Phanerozoic. A wide range of stomatal adaptation to decreasing *CO_2_* during the Phanerozoic is deduced from the fossil record of *s* [µm^2^] and *d* [mm^−2^] evolution [14], [15], [29], [30], providing a unique opportunity to investigate trends in plant gas exchange characteristics across geological time scales during significant changes in atmospheric CO_2_.

## Methodology

### Environmental Conditions During the Phanerozoic

The *CO_2_* time series for the Phanerozoic provided by Berner and Kothvala [27] were used in this study. In contrast with large variations in *CO_2_* during plant evolution*g_w_*evidence suggests that fluctuations in solar radiation and air temperature during the Phanerozoic were relatively minor, with the estimated mean air temperature varying only between 15°C and 23°C [25]. Consequently, variations in potential evaporation, *E_p_*, during plant evolution remained relatively moderate as noted by Sperry [43]: “the maximum gradient for plant water loss to the atmosphere has remained constant during plant evolution”. A simple approximation based on the Penman-Monteith equation for *E_p_* [44] suggests that for reported variation in mean air temperature, *E_p_* would vary by 15%. For simplicity, we assumed a constant *E_p_* value of 7,500 [µmol m^−2^ s^−1^] (corresponding to a mean daily potential evaporation rate of 3 mm d^−1^ and boundary layer thickness δ = 2.0 mm for a mean wind speed of 1 m/s, in accordance with the assumptions in Beerling et al. [12]).

In addition to *CO_2_* levels, the concentration of atmospheric oxygen exhibited fluctuations throughout the Phanerozoic [45], which could affect stomatal function [46]. Nevertheless, we neglected the impact of such fluctuations, which photosynthesis models have shown to have a minor impact on inferences concerning WUE [15].

### Evolution of Stomata Size and Density during the Phanerozoic

**Figure 1:**
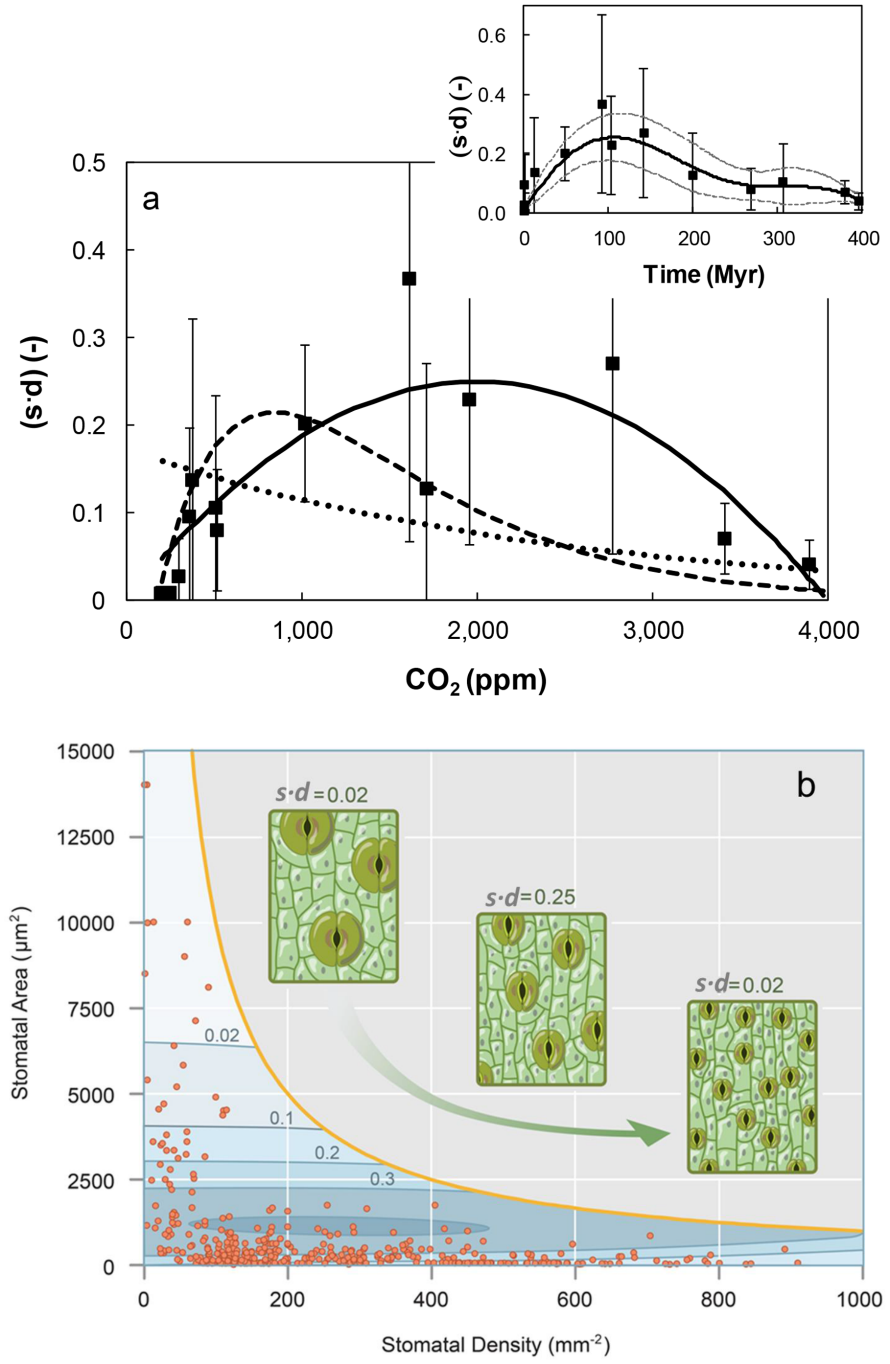
Fossil record-based plant leaf stomatal size and density, (*s* ⋅*d*), during the Phanerozoic. (a) Variations in mean (*s*⋅*d*) with changes in atmospheric CO_2_ concentrations; black symbols represent mean values for different time intervals based on data reported by Franks and Beerling [15]; the solid line is a fitted polynomial to the data (Eq. S1 in Appendix S1); the dashed line represent (*s*⋅*d*) values estimated by the product of Eqs. S3 and S4 from Franks and Beerling [14]; the dotted line represent (*s*⋅*d*) values estimated by the product of Eqs. S5 and S6 from Franks and Beerling [15]. The inset depicts the corresponding change of (*s*⋅*d*) with time (the solid line is the curve fitted using GAM and the upper and lower dashed curves indicate the 95% confidence intervals). (b) Correlated evolution of stomata density *d* [mm^−2^] and area *s* [µm^2^] (symbols) over the Phanerozoic bounded by geometrical maximum (solid line), based on Fig. 1 of Franks and Beerling [14]. Contours of equal (*s*⋅*d*) values highlight the non-monotonic evolution of (*s*⋅*d*) as stomata size decreased and their density increased.

Fossil record data on the evolution of *s* and *d* for a wide range of C3 plants were reported by Franks and Beerling [14]. The *s* and *d* values were averaged for time intervals ranging from 5 to 85 Myr and related to corresponding atmospheric CO_2_ concentrations. The product (*s*⋅*d*) [−], deduced from average values of *s* and *d* for each time interval determines the maximal relative leaf transpiring area, (*a_max_*⋅*d*) [−], and thus strongly influences plant gas exchange. The resulting (*s*⋅*d*) evolution during the Phanerozoic is depicted in Fig. 1a and the fitted expression (solid line) is given in Eq. S1 in Appendix S1. Low values of (*s*⋅*d*) characterize both the beginning of the eon with high *CO_2_* (∼4,000 ppm), and present day values with low *CO_2_* (∼350 ppm). Plant adaptation to decreasing *CO_2_* may be reflected by increasing (*s*⋅*d*) from initial (∼4,000 ppm) to mid-range *CO_2_* levels (∼2,000 ppm), followed by decreasing (*s*⋅*d*) to present day values [30]. The chronological sequence of (*s*⋅*d*) variation during the Phanerozoic is depicted in the inset in Fig. 1a, indicating that approximately 100 Myr ago (corresponding *CO_2_* between 1500 to 2000 ppm) the trend in (*s*⋅*d*) was reversed, and decreased with decreasing *CO_2_*. Statistical analysis including fitting a general additive model (GAM) showed that the trend (solid curve) was highly significant.

Franks and Beerling [14] have used larger time intervals (50 or 100 Myr)for data averaging (resulting in less data points but similar trend), and have fitted regression equations to *s* and *d* individually as functions of *CO_2_* (Eqs. S3 and S4 in Appendix S1). The resulting evolution of (*s*⋅*d*) computed according to their regression equations (Eqs. S3 and S4) is depicted by the dashed line in Fig. 1a. Interestingly, in a related study, Franks and Beerling [15] have reported different trends of *s* and *d* vs. *CO_2_* that yield a monotonously increasing (*s*⋅*d*) with *CO_2_* decrease (dotted line in Fig. 1a). In that analysis of the fossil record, they have used a 10-Myr averaging time interval. The resulting regression equations for *s* and *d* as individual functions of *CO_2_* are presented in Eqs. S5 and S6 in Appendix S1.

The correlated evolution of plant stomata *s* and *d* over the Phanerozoic reflects general transition from large *s-*low *d* configuration (in the early Phanerozoic) to a present day small *s-*high *d* configuration (Fig. 1b). The range of admissible values of *d* and *s* is geometrically limited by the continuous line in Fig. 1b [14], forcing the evolution of *s* and *d* across a non-monotonous (*s*⋅*d*) trajectory (as depicted in Fig. 1a).

Based on Franks and Farquhar [37] and Franks and Beerling [14], the maximal relative transpiring leaf area, (*a_max_*⋅*d*), is proportional to (*s*⋅*d*) (approximated as 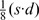. Consequently, the correlated evolution of *s* and *d* is expected to significantly influence plant gas exchange and related attributes *A*, *E*, and WUE.

### Stomatal Configurations Effects on Diffusive Resistances and Transpiration Rate

**Figure 2:**
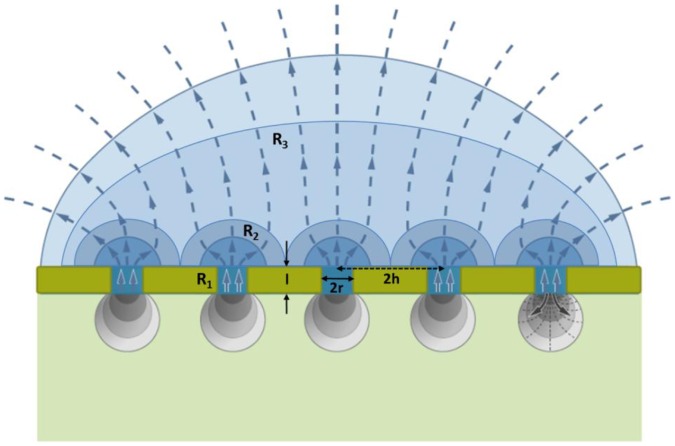
Gas exchange resistances and diffusion fields for water vapor and CO_2_ over plant leaves. Diffusion flow lines (dashed lines) and equal concentrations lines (solid lines) for water vapor in the boundary layer over the leaf (blue areas) and for atmospheric CO_2_ within the substomatal cavities (grey areas) are shown. The figure depicts the consecutive three diffusive resistances resulting from the interactions between neighboring stomata, as expressed in Eqs. (6–9). The schematic representation is adapted from Bange [33].

Diffusion theory predicts formation of a three-dimensional water vapor concentration field over individual stomata as depicted in Fig. (2) [33], [36], [41], [42]. These local vapor “shells” are confined within the boundary layer induced by convective air flow and introduce an additional diffusion resistance term to commonly-used gas exchange models and give rise to nonlinear relationship between evaporative fluxes, *E* [µmol m^−2^ s^−1^], and stomata aperture and density configurations. Studies have distinguished three primary resistances to vapor diffusion from perforated surfaces or plant leaves that operate in series as depicted in Fig. (2) [33]. The first resistance term, *R_1_*, corresponds to the classical single pore resistance [32]; *R_2_*, represents the resistance due to local vapor “shells” forming over pores and reflect interactions among neighboring pores; and *R*_3_, corresponds to the resistance of the boundary layer over the entire membrane or leaf surface. The resistance *R*_3_ is identical to standard resistance, *R_p_*, characterizing vapor diffusion from a reference system (typically free water surface) that represents the potential evaporation, *E_p_*. Several diffusion models have been proposed to quantify these resistances [33], [36], [37], [40]. In this study we adopted the model of Bange [33] based on its modest input parameter requirements and the explicit account for stomatal interactions affecting vapor diffusive fluxes. The model expresses evaporation rate, *E*, across a diffusion barrier perforated by identical pores of radius *r* relative to a potential evaporation rate from uncovered free water surface (under similar conditions), *E_p_*. The first diffusion resistance term, *R*_1_, is defined as:
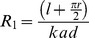
(6)Where *l* is the thickness of the diffusion barrier or membrane [mm], approximated in the case where it represents the stomatal depth by 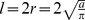; *k*, the ratio of water vapor diffusivity to air molar volume; 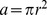, the pore area [mm^2^]; and *d,* the pore density [mm^−2^]. In terms of conductance, Eq. 6 is identical to the classical expression employed to estimation of stomatal conductance to water vapor, *g_ws_* [13], [15]:
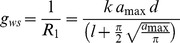
(7)where *a_max_* is the stomata maximum aperture. The second diffusive resistance term, *R_2_*, represents the effect of interactions between neighboring pores or stomata, and it is defined in the model of Bange [33]:
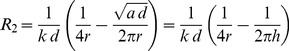
(8)Where *r* is the pore radius [L] and *h*, half mean distance between pores or stomata [L], approximated by 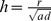. Alternative expressions for *R_2_* proposed by Cooke [37] and Schlünder [40] were compared and yield similar results for the range of (*a_max_⋅d*) values of plant leaves (results not shown).

Finally, the third resistance term, *R*_3_, is expressed by
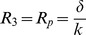
(9)where δ is the thickness of the boundary layer [L] (referred to as viscous sublayer, the region above the surface where the flow is laminar [42]). The three diffusive resistances defined in Eqs. 6 to 9 are expressed per unit of perforated leaf area.

The resulting relative transpiration rate, 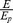, from a leaf surface accounting for the three diffusive resistances is thus:
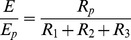
(10)


**Figure 3:**
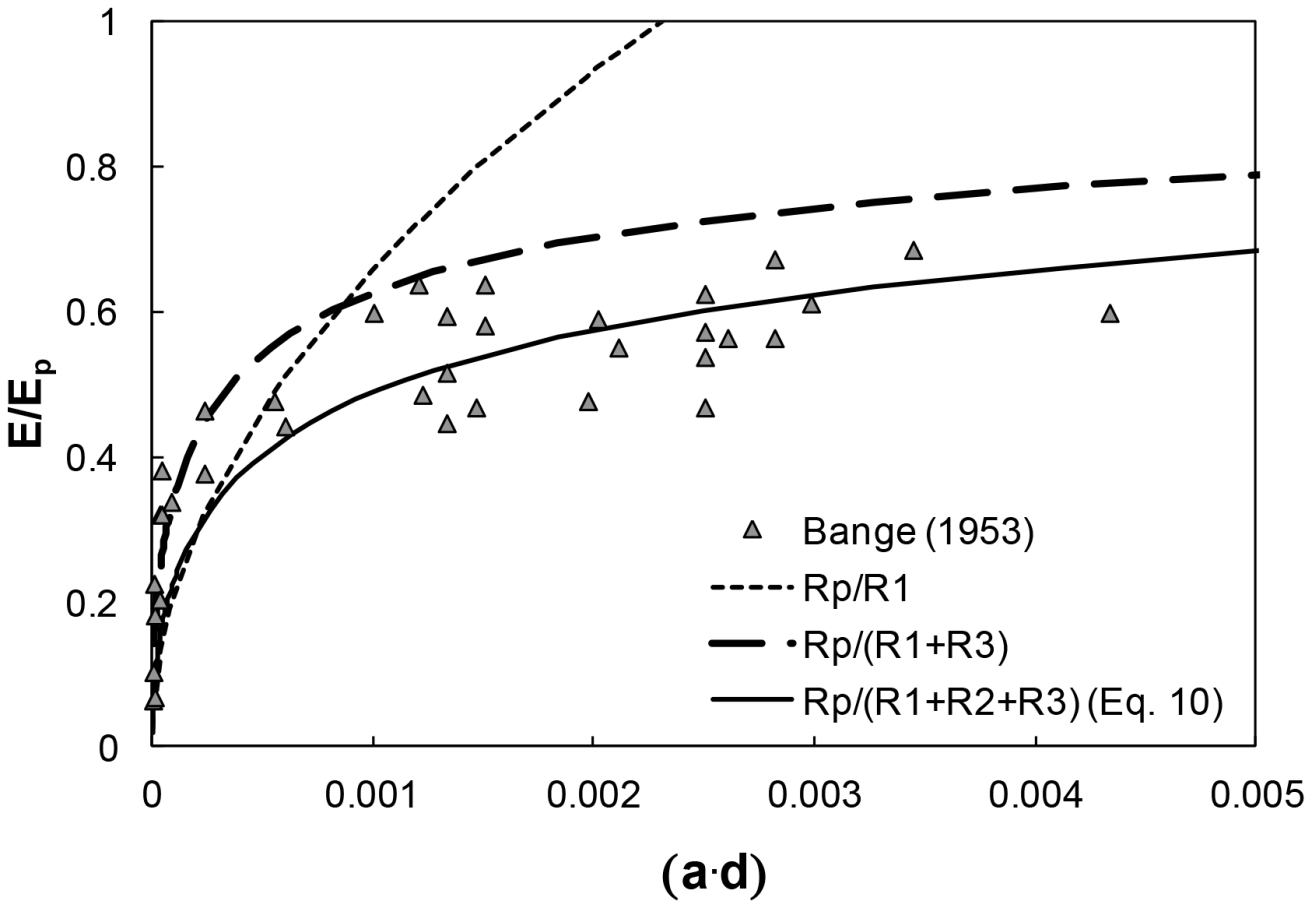
Relative evaporation 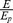 as a function of relative evaporating area (*a*⋅*d*) from *Zebrina pendula* leaves. The symbols depict relative transpiration rates for several stomata apertures ranging from 1 to 20 µm and a mean density of 1625 cm^−2^ based on measurements of Bange [33] in still air. The short-dashed line corresponds to estimates that consider diffusive resistance from single pores only (Eq. 6). The large-dashed line corresponds to estimates that neglect interactions between neighboring stomata. The solid line corresponds to estimates based on Eq. 10 that express the effect of all three resistances depicted in Fig. 2.

The expression in Eq. 10 has been evaluated using relative transpiration rates from *Zebrina pendula* leaves (for a range of stomatal apertures ranging from 1 to 20 µm and similar stomatal density of 1,625 cm^−2^) measured by Bange [33]. The results are depicted in Fig. 3 as a function of the relative transpiring area of the leaf, (*a*⋅*d*). The corresponding boundary layer thickness δ was set to 7.5 mm, well within the range of viscous sub-layer thicknesses expected for air flows according to the Blasius relationship δ∼2v_∞_^−1/2^ with mean air velocity v_∞_ in [m/s], and δ in [mm] [42], [47]. The relative transpiration rate estimated by Eq. 10 that accounts for all three resistance terms was in excellent agreement with measurements for the entire range of experimental values (Fig. 3). In Fig. 3, we also present the relative transpiration rates estimated without consideration of resistances R_2_ and R_3_. The results demonstrate that the resistance R_3_ due to the boundary layer is the most important component of overall resistance to gas diffusion. An important aspect from plant gas exchange point of view, is that consideration of stomatal resistance only, *R_1_* (Eq. 6) does not capture measured fluxes for (*a*⋅*d*) values exceeding 0.001. Neglecting the contribution of interactions between neighboring stomata expressed by R_2_ resulted in overestimation of gas fluxes by 65% for low (*a*⋅*d*) values down to 15% for higher (*a*⋅*d*) values. This suggests that including stomatal interactions systematically reduces gas fluxes with significant effects for certain *a* and *d* combinations, hence such interactions cannot be generally disregarded as suggested by Waggoner and Zetlich [1].

The model in Eq. 10 was applied to estimate the evolution of relative transpiration flux, 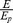 during the Phanerozoic (assuming *E_p_* remained constant as discussed above). For estimation of absolute values of *E*, we have used a constant *E_p_* = 7,500 [µmol m^−2^ s^−1^] equivalent to mean daily potential evaporation rate of 3 mm d^−1^ and boundary layer thickness δ = 2.0 mm that corresponds to mean wind speed of 1 m/s, in accordance with the assumptions in Beerling et al. [12]. A sensitivity analysis indicated that the basic trend deduced in this study was not sensitive to fluctuation in *E_p_* related to variations in mean air temperature (results not shown).

### Estimation of Plant Net CO_2_ Assimilation Rate, *A*

Plant CO_2_ assimilation rate, *A* [µmol m^−2^ s^−1^], is more difficult to estimate than *E*, due to additional assumptions concerning uncertain biochemical processes. We considered two different methods for estimation of *A* as a function of *CO_2_* during the Phanerozoic. Both methods rely on the widely-used photosynthesis model of Farquhar et al. [48] where *A* is expressed as:
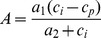
(11)with the constants *a_1_* [µmol m^−2^ s^−1^] and *a_2_* [µmol mol^−1^] whose values depend on light or Rubisco limitations; *c_p_* [µmol mol^−1^] is the CO_2_ compensation point, and *c_i_* the intercellular CO_2_ concentration. For light-saturated conditions, *a_1_* represents the maximum carboxylation capacity, and *a_2_* accounts for both carboxylation rate and the impact of air oxygen concentration on CO_2_ fixation [11]. Following *Katul et al.* [11], *A* is expressed in terms of leaf conductance to CO_2_, *g_c_*, and atmospheric CO_2_ concentration, *c_a_*:



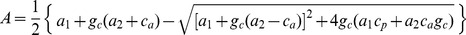
(12)We invoke similarity between diffusive conductance to CO_2_ uptake and water vapor loss (Eq. 2), hence, the leaf conductance *g_c_* considering all diffusive resistances described previously is:
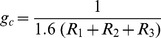
(13)


### Method 1: *V_cmax_* Estimated Following de Boer et al. [16]

Katul et al. [49] proposed parameters and temperature adjustments for the model in Eq. 12, assuming a constant maximal carboxylation capacity at 25°C, *V_cmax_*, [µmol m^−2^ s^−1^] to obtain *a_1_* values. However, evidence suggests that the maximal Rubisco carboxylation rate, *V_cmax_*, decreased with increasing CO_2_ [50]. Therefore, we adopted the model parameters presented in Katul et al. [11] except the assumption concerning a constant *V_cmax_*. Instead we followed de Boer et al. [16] expression of *V_cmax_* as a function of CO_2_:
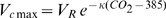
(14a)where *V_R_* is the reference value of *V_cmax_* for a CO_2_ concentration of 385 ppm, and CO_2_ is expressed in [ppm]. Hence, the first method for estimating plant *A* was based on Eqs. 11, 12, 13 and 14a.

### Method 2: *V_cmax_* Estimated Following Franks & Beerling [15]

Beerling and Woodward [29] and Franks & Beerling [15] have developed a coupled photosynthesis-stomatal conductance-energy balance model that also employs the biochemical model of Farquhar et al. [48] for C3 plants. To better account for changes in plant gas conductance (stemming from the evolution of *s* and *d*), Franks and Beerling [15] proposed an empirical relationship expressing *V_cmax_* as a function of gas diffusion conductance *g_w_*:
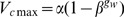
(14b)where *g_w_* is expressed in [mmol m^−2^ s^−1^]. The second method for estimating *A* was based on Eqs. 11, 12, 13 and 14b.

**Figure 4:**
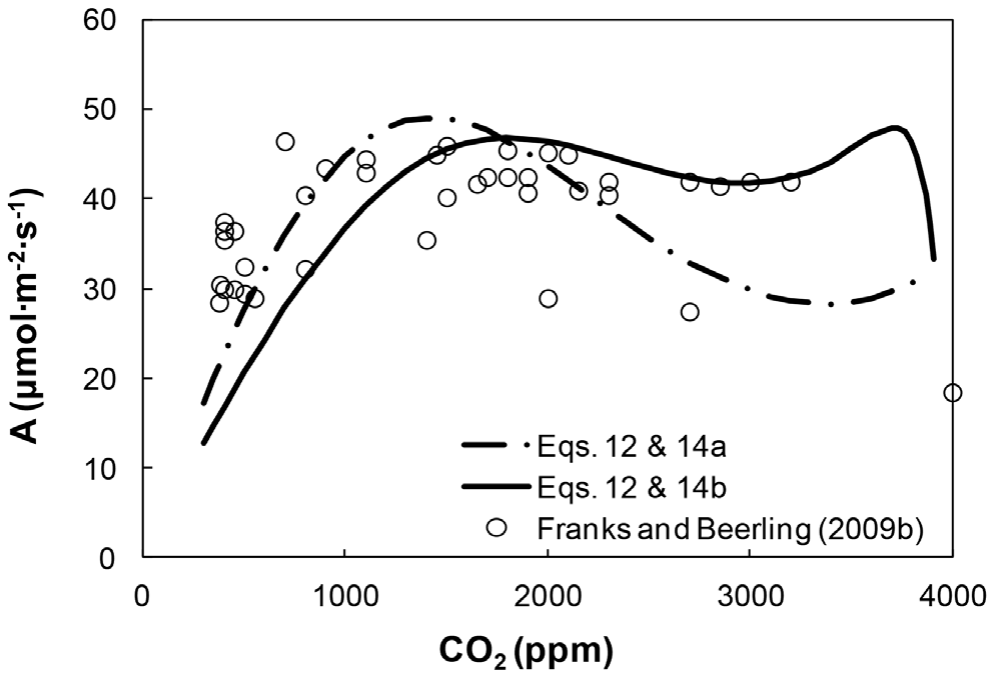
Photosynthetic assimilation rate *A* as a function of atmospheric CO_2_ levels for C3 plants. The curves represent the results from the model of Katul et al. [11, 49] based on [48] (Eq. 12) with *V_cmax_*-*g_w_* given in Eq. 14b (solid line) and *V_cmax_*-*CO_2_* given in Eq. 14a (dashed line). The symbols represent values resulting from the reconstruction of Franks and Beerling [15] (based on results corresponding to the “Upper bound L, variable Temp. and O_2_” case in their Fig. 7A).

Within the unavoidable uncertainties of reconstructing plant *A* for the Phanerozoic, we considered *A* estimates from the reconstruction of Franks and Beerling [15] (symbols), to determine the values of the constants in Eqs. (14a) and (14b). The best agreement for *A*-*CO_2_* relationships was obtained for the following parameter values: *V_R_* = 120 µmol m^−2^ s^−1^; κ = 2.8 10^−4^ ppm^−1^; α = 82 µmol m^−2^ s^−1^; β = 0.974. The *A*-*CO_2_* relationships resulting from the two methods applied are depicted in Fig. 4 (curves) as functions of atmospheric CO_2_ concentrations, along with the estimates of *A* as reported in figure 7A (symbols) of Franks and Beerling [15]. The time course of *A* estimates is similar to that described in that figure in Franks and Beerling [15].

## Results and Discussion

### Evolution of Plant WUE during the Phanerozoic

**Figure 5:**
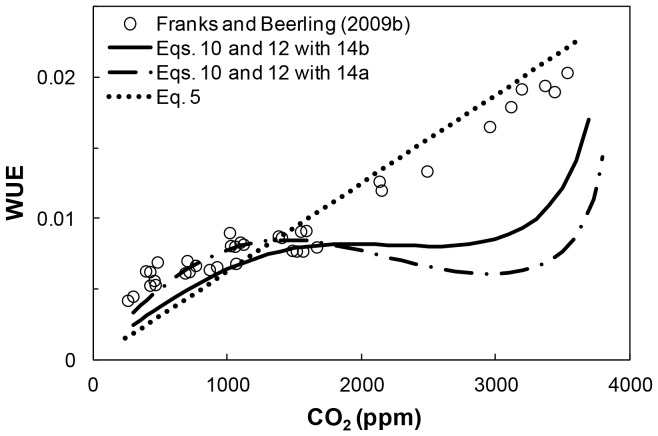
Reconstructed WUE-*CO_2_* relationship for C3 plants during the Phanerozoic. The curves result from the independent estimates of *A* based on Eq. 12 with Eq. 14a (dashed line) and Eq. 14b (solid line), and *E* (Eq. 10; with *E_p_* = 7500 [µmol m^−2^ s^−1^] and δ = 2.0 mm) for the parabolic (*s*⋅*d*)-*CO_2_* relationship (Eq. A1).The dotted line corresponds to WUE estimates based on Eq. 5, with *p_i_*/*p_a_* = 0.7 and Δ*e* = 0.03. The symbols represent values resulting from the model of Franks and Beerling [15] (based on their Fig. 10).

Plant WUE (Eq. 1) was evaluated from independent estimates of *A* and *E* (Eqs. 12 and 10), and is expressed as a function of *CO_2_* in Fig. 5 (solid and dashed lines for the two methods for *A* estimation described above). The resulting WUE-*CO_2_* trends were compared with the conventional approach (Eq. 5; dotted line in Fig. 5), and with modeled WUE by Franks and Beerling ([15] (symbols in Fig. 5). The latter two depict a quasi-linear decreasing trend for WUE-*CO_2_*. In contrast, the estimated plant WUE trend resulting from independent estimates of *E* and *A* exhibit a complex behavior. Results suggest that plant gas exchange adaptation resulted in a steep decrease in WUE in response to the initial drop in *CO_2_*, followed by leveling off to *CO_2_* values of ∼1,200 ppm; and subsequently WUE decreased as *CO_2_* declined to present day values. Interestingly, for the last period of plant WUE evolution, all models converge and yield a similar WUE decreasing trend.

At first glance, the interpretation of the conventional nearly-linear decreasing WUE with declining *CO_2_* would imply that improving CO_2_ uptake was the primary driver for plant gas exchange evolution. However, consideration of the new WUE trend (Fig. 5; solid line) shows that plant WUE remained practically constant despite the large decrease in *CO_2_* (from 3,500 to 1,200 ppm). This trend lends support to an alternate interpretation whereby regulation of water losses and not improving CO_2_ uptake could have served as the primary driver for plant gas exchange evolution, especially during a period where plants have gradually adapted to life in dryer terrestrial environments [5], [8].

### Estimating Plant Transpiration, *E*, during the Phanerozoic

**Figure 6:**
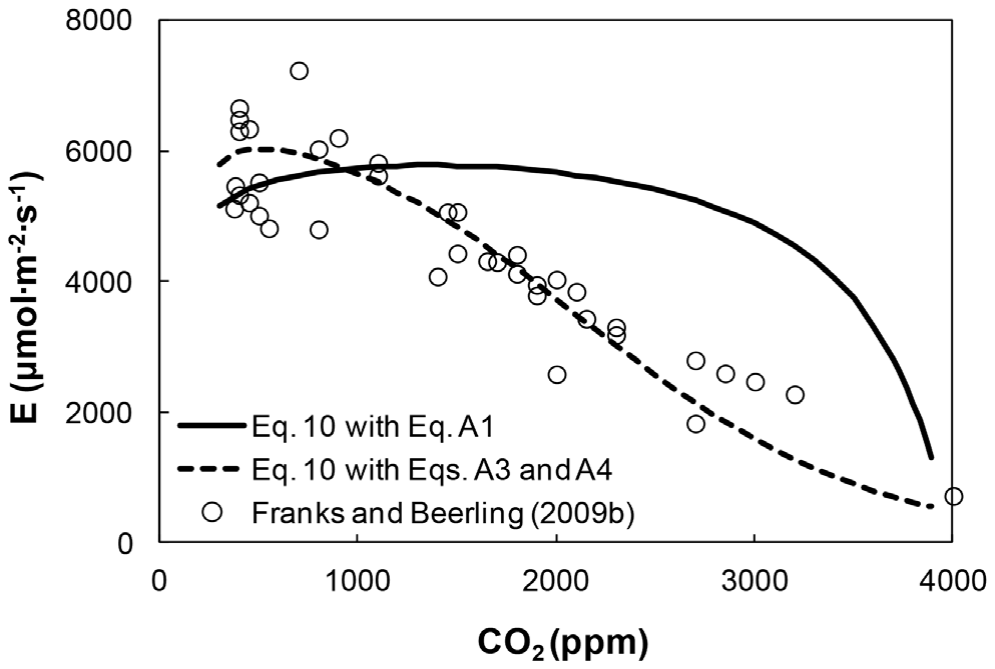
Relationship between transpiration rate *E* and *CO_2_* for C3 plants during the Phanerozoic. The solid line corresponds to Eq. 10 using the parabolic (*s*⋅*d*)**-** *CO_2_* relationship in Eq. S1, and the dashed line corresponds to the skewed (*s*⋅*d*)**-** *CO_2_* relationship resulting from Eqs. S3 and S4. The symbols are computed from the ratio of modeled values of WUE and *A* in [15].

Plant transpiration rate, *E,* was estimated based on Eq. 10, considering resistive terms (Eqs. 6–9) computed for specific *a_max_–d* configurations at each *CO_2_* level corresponding to the *s-d* patterns in Fig. 1a (solid and dashed lines). The respective *E*-*CO_2_* relationships are depicted in Fig. 6 (solid line and dashed line). The symbols in Fig. 6 represent *E* values deduced from the ratio of WUE and *A* values (represented by the symbols in Figs. 5 and 4, respectively) from the model results in [15]. The *E*-*CO_2_* relationship for the skewed (*s*⋅*d*)-*CO_2_* function based on Franks and Beerling [14] empirical relations (dashed line in Fig. 1*a*), and the values extracted from the model of Franks and Beerling [15] both exhibit a nearly-linear increase in *E* with decreasing *CO_2_*. In contrast, the *E*-*CO_2_* relationship based on Eq. 10 (with the solid (*s*⋅*d*)-*CO_2_* line in Fig. 1*a*) yields nearly constant plant transpiration rates for most of the range of *CO_2_* changes during the Phanerozoic. The initial steep increase of *E* is associated with increasing (*s*⋅*d*) as *CO_2_* decreased early in the Phanerozoic (Fig. 1a). These results suggest that evolutionary refinements in *s*-*d* configurations enabled plants to maintain a relatively constant transpiration rate for a wide range of *CO_2_* levels.

The results in Fig. 6 highlight the role of stomatal configuration in shaping plant *E*. The gradual increase in (*s*⋅*d*) with decreasing *CO_2_* for the rising limb of the skewed (*s*⋅*d*)-*CO_2_* relationship (Fig. 1a; dashed line) resulted in a nearly-linear increase in *E*, whereas for the parabolic-shaped relation (Fig. 1a; solid line), *E* increased nonlinearly. Interestingly, the falling limbs of both (*s*⋅*d*)-*CO_2_* relationships exerted only a small impact on the *E*-*CO_2_* trend. The largest influence on plant gas exchange seems to be associated with the early increase in (*s*⋅*d*) reflecting a gradual shift from large *s*-low *d* to small *s*-high *d* configuration (Fig. 1b), that correspond to large *a_max_*-low *d* and small *a_max_*-high *d* configuration. The resulting small *a_max_*-high *d* configuration represents a more robust configuration that greatly reduced sensitivity of diffusive gas exchange to reduced leaf evaporating area (*a_max_⋅d*).

We note that in contrast with the steep decrease in *A* for *CO_2_* concentrations below 1,200 ppm (Fig. 4), *E* remained practically constant for the same period (and for the corresponding changes in *CO_2_*) as seen in Fig. 6. These diverging responses of *A* and *E* suggest that total resistances for plant CO_2_ uptake and water loss are different. Part of this difference are attributed to additional biochemical/photosynthetic resistance that affect CO_2_ uptake but not water loss, and to other factors not explored here such as mesophyll resistance [51], [52] and cell walls resistances [12]. Consequently, plant response to environmental changes in terms of *A* may be different and decoupled from the response in plant *E*. Additional factors involving asymmetry in diffusion fields for water vapor and CO2 have been discussed by Parkhurst [53].

### Variations in Plant *A* and *E* with Evolution of Stomata *s* and *d*

**Figure 7:**
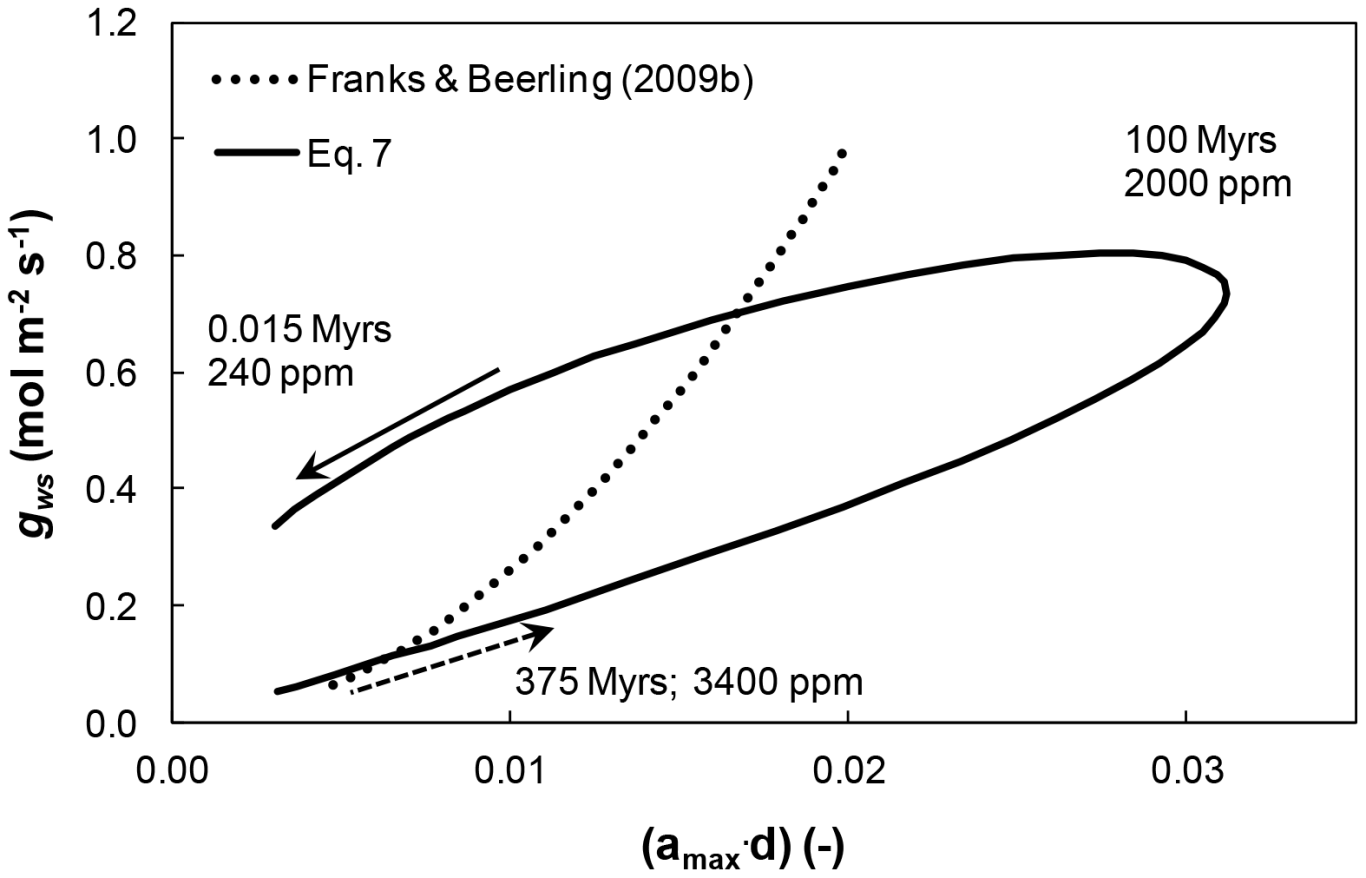
Relationship between maximal vapor diffusive conductance of a stoma and maximal leaf partial transpiring area. Estimated variations in maximal vapor diffusive conductance of a stoma, *g_ws_* (Eq. 7) [mol m^−2^ s^−1^] and maximal leaf partial transpiring area (*a_max_*⋅*d*) based on values from Eqs. (S1) and (S2) (solid line). The dotted line corresponds to *g_ws_*-(*a_max_*⋅*d*) computed according to Eq. 7 with the regression equations of Franks and Beerling [15] (Eqs. S5 and S6). Arrows depict chronology (dashed arrows represent early Phanerozoic; solid arrows represent late Phanerozoic).

The impact of *s* and *d* evolution on maximal conductance to water vapor, *g_ws_* [mol m^−2^ s^−1^], was estimated based on Eq. 7, using the *a_max_* and *d* values corresponding to the *s* and *d* values in Eqs. S1 and S2, and those of Franks and Beerling [15] in Eqs. S5 and S6. The evolution of *g_ws_* as a function of (*a_max_*⋅*d*) variations during the Phanerozoic is depicted in Fig. 7. The resulting hysteretic-like behavior of *g_ws_*-(*a_max_*⋅*d*) reflects the parabolic (*s*⋅*d*)-*CO_2_* relationship (Fig. 1a; solid line), and differs from the monotonously increasing *g_ws_*-(*a_max_*⋅*d*) relation resulting from the monotonous (*s*⋅*d*)-*CO_2_* relationship (Fig. 1a; dotted line). The non-monotonous behavior suggests that plant adjustment of stomatal configuration by selection for small *a_max_-*high *d* resulted in higher *g_ws_* for similar (*a_max_*⋅*d*) values. The two trends in Fig. 7 suggest that stomatal evolution has led to increased diffusive gas conductance. Hence, the shift towards small *a_max_-*high *d* configuration not only enabled lower diffusive gas resistance with lower (*a_max_*⋅*d*), it also reduced gas exchange sensitivity to leaf evaporating area (as evidenced by maintenance of nearly constant transpiration rates). It may have also contributed to improved leaf thermal management [8], [12].

**Figure 8:**
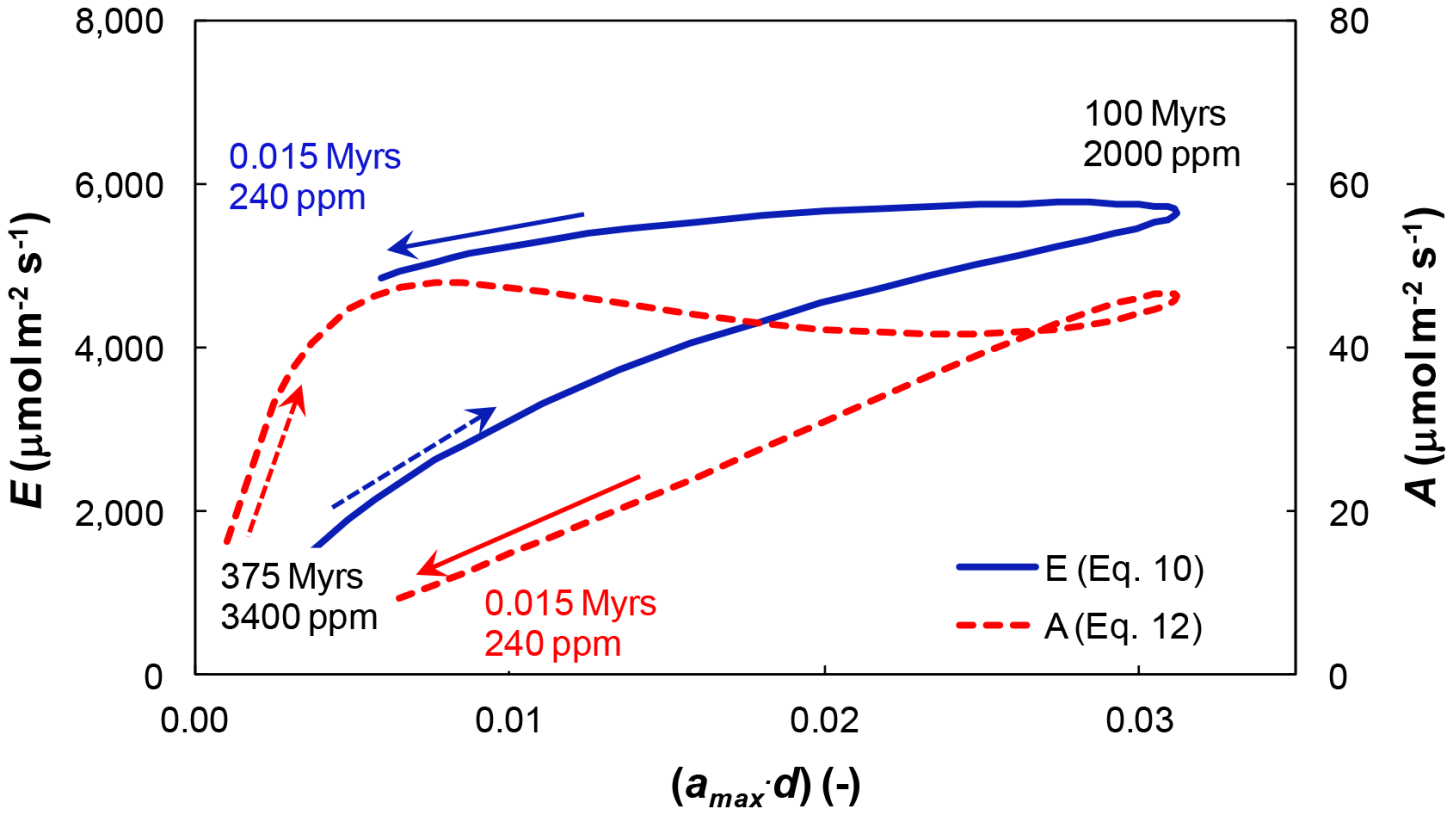
Non-unique relationships between transpiration and CO_2_ assimilation rates and maximal leaf partial evaporative area. Computed relationships between maximal leaf partial evaporative area (*a_max_*⋅*d*) and transpiration rate, *E*, according to Eq. (10) (solid line), and between (*a_max_*⋅*d*) and CO_2_ assimilation rate, *A*, according to Eq. (12) (dashed line) for the parabolic (*s*⋅*d*)-CO_2_ function (Eq. S1). Arrows depict chronology (dashed arrows represent early Phanerozoic; solid arrows represent late Phanerozoic).

Figure 8 depicts the evolution of transpiration rates (Eq. 10) and CO_2_ assimilation rates (Eq. 12) as functions of leaf partial evaporative area (*a_max_*⋅*d*) for the parabolic (*s*⋅*d*)-*CO_2_* relationships estimated in this study (Fig. 1a; solid line). The hysteretic-like behavior of *g_ws_*-(*a_max_*⋅*d*) (Fig. 7) is reflected in both *E*-(*a_max_*⋅*d*) and *A*-(*a_max_*⋅*d*) (Fig. 8). Surprisingly, the trends exhibited by the hysteretic-like behavior of *E*-(*a_max_*⋅*d*) and *A*-(*a_max_*⋅*d*) differ significantly after the hysteretic apex. Following the joint initial increase in *E* and *A* with increasing plant (*a_max_*⋅*d*) (see Figs. 4 and 6), a subsequent decrease in (*a_max_*⋅*d*) resulted in a virtually stable *E* whereas *A* exhibited a consistent decrease. The decrease in (*a_max_*⋅*d*) was accompanied by the selection for smaller and denser stomata (Fig. 1b), which reduced gas resistance for lower leaf evaporating area (Fig. 7). Consequently, *E* remained constant as (*a_max_*⋅*d*) decreased to present day values. These improvements in diffusive gas exchange were insufficient to compensate for the decrease in *CO_2_*, hence *A* continually decreased with decreasing (*a_max_*⋅*d*).

### Summary and Conclusions

We have considered *s* and *d* data from plant fossil record spanning the Phanerozoic eon to investigate effects of stomatal configuration on plant CO_2_ uptake, *A*, on transpiration, *E*, rates, and on plant water use efficiency, WUE. Plant transpiration rates were estimated based on the diffusion resistance model of Bange [33], whereas CO_2_ uptake rates were estimated based on the model of Farquhar et al. [48]. Both gas fluxes considered stomata interactions in the leaf gas resistance formulation. Different (*s*⋅*d*)-*CO_2_* patterns resulting from different analyzes of the fossil record were considered. Stomata configuration, expressed in terms of maximal relative leaf evaporating area, (*a_max_*⋅*d*), was found to affect mostly the gas exchange rates during the first phase of the Phanerozoic when large *s*-low *d* configuration prevailed. It is likely that the drastic drop in atmospheric CO_2_ induced a shift from large *s*-low *d* configuration to small *s*-high *d* configuration. This shift enabled lower diffusive gas resistance with lower (*a_max_*⋅*d*), decreased gas exchange sensitivity to leaf evaporating area and may has also contributed to improved leaf thermal management.

The estimated plant WUE during the Phanerozoic reveals a significantly different trend than the conventional WUE postulating proportionality with atmospheric CO_2_ concentration. In contrast with the linear decrease in WUE with decreasing *CO_2_*, the estimated WUE was relatively stable during a significant part of the eon. This new trend results from decoupled response of plant *A* and *E* to the correlated evolution of *s* and *d* and their related product (*a_max_*⋅*d*). We observed that the shape of *A* exerts a stronger influence on the WUE pattern than the shape of *E*. Uncertainties in past plant biochemical photosynthetic response introduces complexities into the estimation of *A*, and thus in estimates of WUE. Nevertheless, the new WUE trend was relatively robust using two variations in the model for *A.* This trend suggests that regulation of water losses could have served as the primary driver for plant gas exchange evolution, thus contributing to the invasion of dryer terrestrial environments [5], [8].

## Supporting Information

Appendix S1 **Detailed presentation of the three different methods used to quantify the changes in stomatal density,** ***d*** **, and size,** ***s*** **with atmospheric CO_2_ concentrations during the Phanerozoic eon based on fossil record.** These three methods led to the three curves depicted in Fig. 1a describing the resulting (*s*⋅*d*) evolution during the Phanerozoic.
(DOCX)
