# Correction: Plant Water Use Efficiency over Geological Time—Evolution of Leaf Stomata Configurations Affecting Plant Gas Exchange

**DOI:** 10.1371/journal.pone.0127015

**Published:** 2015-04-27

**Authors:** Shmuel Assouline, Dani Or

There are errors in Eq 6 and its description. Please view the complete, correct equation here:

$$R_{1}=\frac{l}{k\;a\;d}$$

where *l* is the thickness of the diffusion barrier or membrane [mm], approximated in the case where it represents the stomatal depth by $l=r=\sqrt{\frac{a}{\pi }}$; *k*, the ratio of water vapor diffusivity to air molar volume; $a=\pi r^{2}$, the pore area [mm^2^]; and *d*, the pore density [mm^-2^].

There are errors in Eq 7 and its description. Please view the complete, correct equation here:

$$g_{ws}=\frac{1}{R_{ws}}=\frac{k\;a_{\max}\;d}{\left(l+\frac{\pi }{2}\sqrt{\frac{a_{\max}}{\pi }}\right)}$$

where *a*_*max*_ is the stomata maximum aperture, and $R_{ws}=R_{1}+\frac{1}{2k\;r\;d}$, which accounts for the addition of two end corrections to the pore resistance R_1_ [33].

There are errors in Fig 3 and its legend. Please see the corrected Fig 3 here.

**Fig 3:**
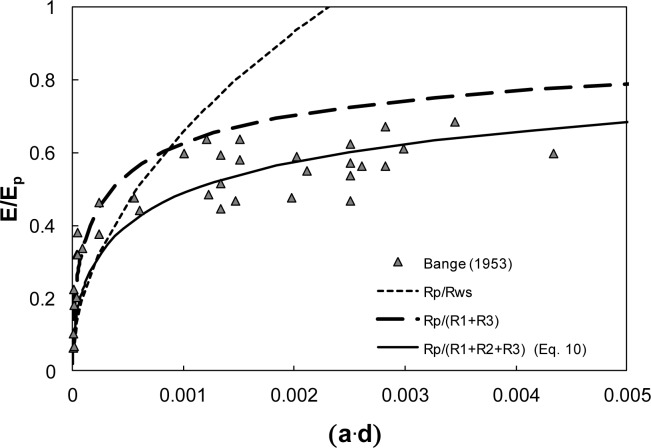
Relative evaporation $\frac{E}{E_{p}}$ as a function of relative evaporating area *(a-d)* from *Zebrina pendula* leaves. The symbols depict relative transpiration rates for several stomata apertures ranging from 1 to 20μm and a mean density of 1625 cm^-2^ based on measurements of Bange [33] in still air. The short-dashed line corresponds to estimates that consider diffusive resistance from single pores only (Eq 7). The large-dashed line corresponds to estimates that neglect interactions between neighboring stomata. The solid line corresponds to estimates based on Eq 10 that express the effect of all three resistances depicted in Fig 2.
