# Supplementary material for: Plant Water Use Efficiency over Geological Time – Evolution of Leaf Stomata Configurations Affecting Plant Gas Exchange
Source: PLoS One. 2013 Jul 2;8(7):e67757. doi: 10.1371/journal.pone.0067757 (PMC3699479; doi:10.1371/journal.pone.0067757)
Supplement: Appendix S1 — Detailed presentation of the three different methods used to quantify the changes in stomatal density, d , and size, s with atmospheric CO2 concentrations during the Phanerozoic eon based on fossil record. These three methods led to the three curves depicted in Fig. 1a describing the resulting (s⋅d) evolution during the Phanerozoic. (DOCX) [file pone.0067757.s001.docx]

**Appendix S1**

Plant fossil record revealed that plants adaptation to changing conditions during the Phanerozoic involved significant changes in stomatal density, *d*, and size, *s* [14, 15, 29, 30].

In this study, the *s* and *d* values were averaged for time intervals ranging from 5 to 85 Myr. Plant gas exchange rates being determined by stomatal configuration expressed in terms of the product between *s* and *d*, *(s·d)*, the following regression equations were fitted to the data:

(*s^.^d*)=-6.5 10^-8^ [*CO_2_*]^2^ + 2.55 10^-4^ [*CO_2_*] ; *r*^2^=0.70 (S1)

*s* = 1.77[CO_2_] – 14.93 ; *r*^2^=0.83 (S2)

where CO_2_ is given in [ppm]; *(s·d)* in Eq. (S1) is dimensionless*,* and *s* in Eq. (S2) is given in [μm^2^] with. The coefficients in Eq. S1 were significant at the level of *p<0.05*. The (*s^.^d*)- *CO_2_* relationship (Eq. S1) is depicted by the solid line in Fig. 1a.

In their analysis of the fossil record, Franks and Beerling [14] considered time intervals of 50 or 100 Myr. They have fitted regression equations to the mean values of *s* and *d* for each time interval:

log_10_ *d* = -6.57 10^-4^ [CO_2_] + 2.58 ; *r*^2^=0.98 (S3)

*s* = 3.02[CO_2_] – 528 ; *r*^2^=0.92 (S4)

where *d* ins given in [mm^-2^], *s* in [μm^2^] and CO_2_ in [ppm]. Based on Eqs. S3 and S4, the resulting evolution of (*s^.^d*) with *CO_2_* was computed and depicted by the dashed line in Fig. 1a.

Franks and Beerling [15] considered time intervals of 10 Myr. They have fitted regression curves to the mean values of *s* and *d* for each time interval:

log_10_ *d* = -5.08 10^-4^ [CO_2_] +2.39 ; *r*^2^=0.57 (S5)

log_10_ *s* = -0.65 log_10_ *d* +4.4 ; *r*^2^=0.45 (S6)

where *d* is given in [mm^-2^], *s* in [μm^2^] and CO_2_ in [ppm]. Based on Eqs. S5 and S6, the resulting evolution of (*s^.^d*) with *CO_2_* was computed and depicted by the dotted line in Fig. 1a.
